# Radiomics for preoperative prediction of early recurrence in hepatocellular carcinoma: a meta-analysis

**DOI:** 10.3389/fonc.2023.1114983

**Published:** 2023-06-07

**Authors:** Huan Tian, Yong Xie, Zhiqun Wang

**Affiliations:** ^1^Department of Radiology, Aerospace Center Hospital, Beijing, China; ^2^Department of Interventional Radiology and Vascular Surgery, Peking University First Hospital, Beijing, China

**Keywords:** hepatocellular carcinoma, radiomics, early recurrence, prediction, sensitivity, specificity, meta-analysis

## Abstract

**Background/Objective:**

Early recurrence (ER) affects the long-term survival prognosis of patients with hepatocellular carcinoma (HCC). Many previous studies have utilized CT/MRI-based radiomics to predict ER after radical treatment, achieving high predictive value. However, the diagnostic performance of radiomics for the preoperative identification of ER remains uncertain. Therefore, we aimed to perform a meta-analysis to investigate the predictive performance of radiomics for ER in HCC.

**Methods:**

A systematic literature search was conducted in PubMed, Web of Science (including MEDLINE), EMBASE and the Cochrane Central Register of Controlled Trials to identify studies that utilized radiomics methods to assess ER in HCC. Data were extracted and quality assessed for retrieved studies. Statistical analyses included pooled data, tests for heterogeneity, and publication bias. Meta-regression and subgroup analyses were performed to investigate potential sources of heterogeneity.

**Results:**

The analysis included fifteen studies involving 3,281 patients focusing on preoperative CT/MRI-based radiomics for the prediction of ER in HCC. The combined sensitivity, specificity, and area under the curve (AUC) of the receiver operating characteristic were 75% (95% CI: 65-82), 78% (95% CI: 68-85), and 83% (95% CI: 79-86), respectively. The combined positive likelihood ratio, negative likelihood ratio, diagnostic score, and diagnostic odds ratio were 3.35 (95% CI: 2.41-4.65), 0.33 (95% CI: 0.25-0.43), 2.33 (95% CI: 1.91-2.75), and 10.29 (95% CI: 6.79-15.61), respectively. Substantial heterogeneity was observed among the studies (I²=99%; 95% CI: 99-100). Meta-regression showed imaging equipment contributed to the heterogeneity of specificity in subgroup analysis (*P*= 0.03).

**Conclusion:**

Preoperative CT/MRI-based radiomics appears to be a promising and non-invasive predictive approach with moderate ER recognition performance.

## Introduction

Hepatocellular carcinoma (HCC) is one of the most common malignant tumors in the world, and the incidence and mortality rate of the disease in the world is increasing year by year (1–3). At present, the situation that HCC patients in China account for nearly half of the world’s total population is not optimistic, which has brought great challenges to the healthcare system and a substantial socioeconomic burden in China (4, 5).

Currently, the radical methods of HCC are still radical resection, liver transplantation, ablation, and other treatments such as vascular intervention have also achieved satisfactory results (6). However, no matter which method is used, some patients still experience poor efficacy and a certain rate of early recurrence (ER) (21.9%-70.2%) after HCC treatment (7, 8).

ER following radical treatment for HCC is defined as intrahepatic or extrahepatic recurrence within one to two years (9, 10). ER is a significant factor affecting HCC prognosis and disease outcome (11). Thus, the identification of sensitive markers associated with ER of HCCs is crucial in facilitating accurate prognostic classification and timely interventions which, in turn, improve overall survival rates. Fortunately, in recent years, radiomics has demonstrated a unique advantage (12). By extracting significant amounts of image information from computed tomography (CT) and magnetic resonance imaging (MRI) with high throughput, it facilitates tumor segmentation, feature extraction, and model establishment. Radiomics enables clinicians to analyze vast image data information in-depth, thereby assisting them in making more accurate diagnoses and predictions.

Many previous studies have utilized CT/MRI-based radiomics to predict ER after radical treatment, achieving high predictive value (7, 9, 10, 13, 14). However, the reported results seem quite variable due to the fact that these above studies differed in the diagnostic performance of the preoperative evaluation of ER because the differences in imaging modalities, research methods, sample size, etc. For these reasons, the diagnostic performance of radiomics for the preoperative identification of ER remains uncertain. Therefore, we performed this meta-analysis to investigate the predictive performance of preoperative CT/MRI-based radiomics for ER in HCC.

## Methods

This study was conducted strictly according to the Preferred Reporting Items for Systematic reviews and Meta-Analysis of Diagnostic Test Accuracy (PRISMA-DTA) (15).

### Retrieval strategy and study selection

In this study, the following Medical Subject Headings (MeSH) and their variations were used in PubMed, Web of Science (all databases including MEDLINE), EMBASE and the Cochrane Central Register of Controlled Trials from inception to December 24, 2022: (“hepatocellular carcinoma” OR “carcinoma, hepatocellular” OR “HCC”) AND (“CT”, “computed tomography”) AND (“diagnosis” OR “accuracy” OR “specificity” OR “sensitivity”) AND (“MRI” OR “magnetic resonance imaging”) AND (“Radiomic*”). PubMed’s search strategy was as follows: ((“hepatocellular carcinoma”[Title/Abstract] OR “carcinoma hepatocellular”[Title/Abstract] OR “HCC”[Title/Abstract]) AND (“diagnosis”[Title/Abstract] OR “accuracy”[Title/Abstract] OR “specificity”[Title/Abstract] OR “sensitivity”[Title/Abstract]) AND (“CT”[Title/Abstract] OR “computed tomography”[Title/Abstract]) AND “radiomic*”[Title/Abstract]) OR ((“hepatocellular carcinoma”[Title/Abstract] OR “carcinoma hepatocellular”[Title/Abstract] OR “HCC”[Title/Abstract]) AND (“diagnosis”[Title/Abstract] OR “accuracy”[Title/Abstract] OR “specificity”[Title/Abstract] OR “sensitivity”[Title/Abstract]) AND (“MRI”[Title/Abstract] OR “magnetic resonance imaging”[Title/Abstract]) AND “radiomic*”[Title/Abstract]). There are no language restrictions in the literature search process.

The inclusion criteria were as follows: we included all eligible radiomics articles that used CT or MRI to assess ER (which was defined as the presence of new intrahepatic lesions or metastasis with typical imaging features of HCC (who experienced radical resection, radical ablation, and liver transplantation), or atypical findings with histopathological confirmation within 2 years) in patients with HCC. The exclusion criteria were as follows: (1) the studies did not have enough information to construct a two-by-two contingency table; (2) antitumor therapy was performed preoperatively; (3) academic review, conference abstracts, animal experiments, expert opinions, books, nondiagnostic tests, guidelines, and case report.

### Data extraction and quality assessment

Two independent researchers (HT and YX) (radiologists with 6 and 5 years of experience) extracted data from the included studies: first author, publication year, study period, study design (prospective or retrospective), cohort detail, demographic characteristics of the study population (such as age, gender), sample size, comorbid conditions, Tumor information (diameter, number, margin, etc.), true positive (TP), false positive (FP), false negative (FN), and true negative (TN), interval from post-operation to early recurrence, CT/MRI imaging equipment, contrast agent, treatment, MRI sequence, radiomics features extracting software. Inconsistencies between the two investigators are resolved through discussion or consultation with the senior investigator.

We evaluated the methodological quality of the included studies by using the standard Quality Assessment of Diagnostic Accuracy Studies-2 (QUADAS-2) tool (Bristol University, Bristol, UK) (16). We assessed the risk of bias and concerns regarding applicability by the software Review Manager 5.4 (Cochrane Library Software, Oxford, UK; available at https://training.cochrane.org/online-learning/core-software/revman). The four aspects of the evaluation are as follows: patient selection, index test, reference standard, and flow and timing. Risk of bias was classified as ‘yes’, ‘no’ or ‘unclear’ and applicability ‘high’, ‘low’ or ‘unclear’. Furthermore, radiomics quality score (RQS) were used to assess the quality of radiomics studies (17).

### Statistical analysis

In this study, Stata17.0 (Stata Corp LP, College Station, Texas, SA) was used for all statistical calculation. We used a bivariate regression model to calculate the combined sensitivity, specificity, positive likelihood ratio (PLR), negative likelihood ratio (NLR), diagnostic score, and diagnostic odds ratio (DOR) with their corresponding 95% confidence intervals (CI), all of which are shown in forest plots. We plotted the pooled results onto the summary receiver operating characteristic (SROC) curve and determined the area under the receiver operating characteristic curve (AUC) to reflect the diagnostic power of the included studies. In addition, the Stata Midas module was used to detect the magnitude of heterogeneity due to threshold effects. The threshold effect test was obtained by calculating the spearman correlation coefficient of the logarithm of sensitivity to the logarithm of (1-specificity), and if *P* is less than 0.05, a threshold effect is present. Heterogeneity caused by non-threshold effects was measured using Cochrane’s Q-test and inconsistency index I^2^, and the difference was considered significant when *P <*0.05, with I^2^ ≥ 50% regarded as being indicative of moderate-to-high heterogeneity among studies (18). In addition, we plotted Fagan nomogram through the Midas module, and post-test probabilities were calculated by pre-test probabilities (the ratio of ER-positive cases to all cases in the included studies), PLR, and NLR. Furthermore, publication bias was assessed by using the Deeks asymmetric regression test. Finally, meta-regression and subgroup analysis were used to explore potential sources of heterogeneity. All hypothesis tests were statistically significant with *P <*0.05 (two-sided).

## Results

### Literature selection

**Figure 1:**
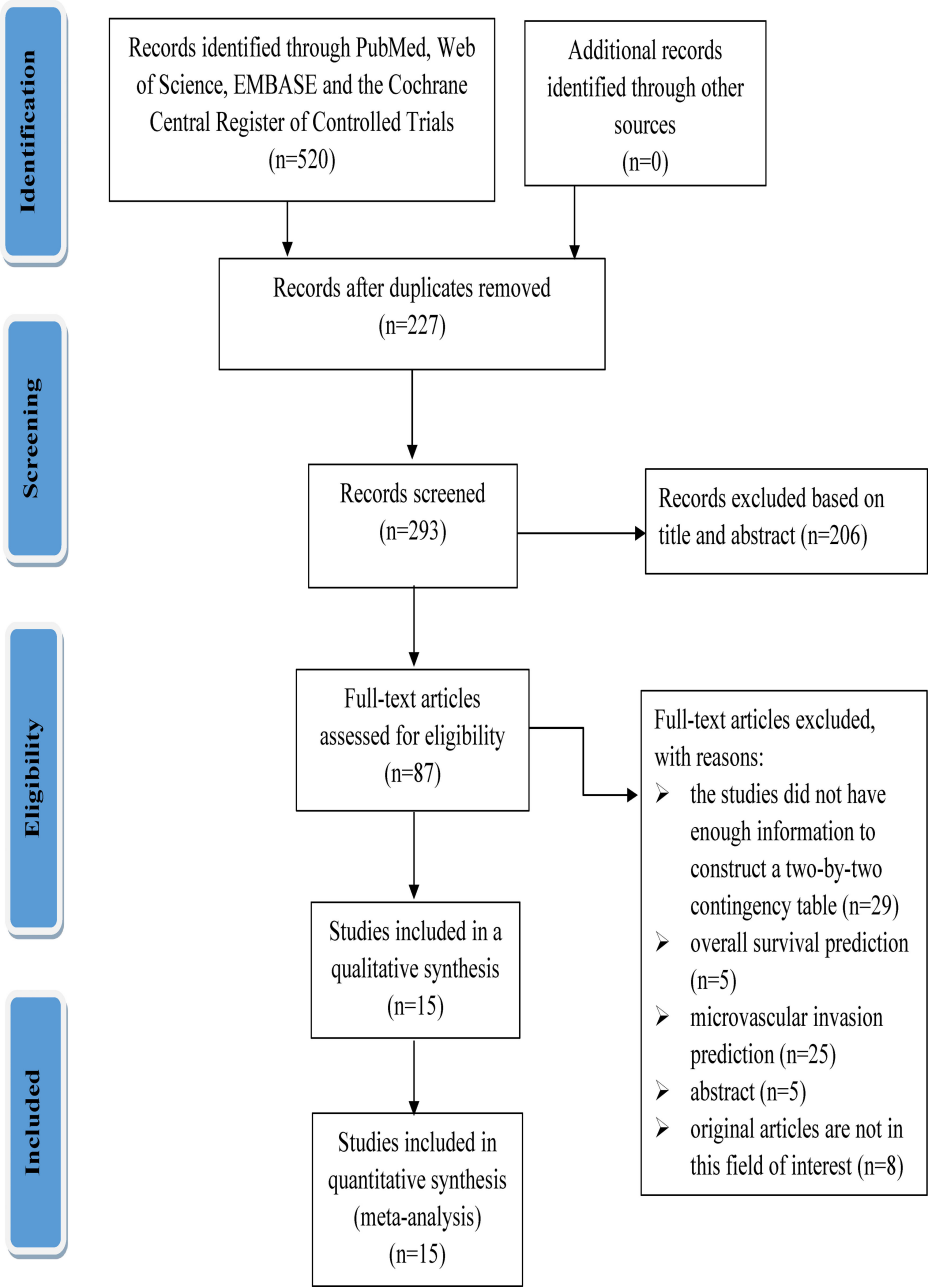
PRISMA flowchart for study selection.

A total of 520 relevant literature were generated through the systematic search. The process of literature search and study selection is presented in **Figure 1**. After screening according to the pre-defined inclusion/exclusion criteria, a total of 15 publications were identified for this meta-analysis, consisting of six radiomics studies based on CT and nine radiomics studies based on MRI (7, 9, 10, 13, 14, 19–28). Most of the studies included in the analysis were retrospective, and all of them were published between 2017 and 2022. The analysis included a total of 3,281 patients with hepatocellular carcinoma. The characteristics of these studies are presented in **Tables 1**, **2**.

**Table 1:** Basic characteristics of the included studies.

First author and year	Design	Time period	Cohort detail	Sample size	Age (mean ± SD or median)	Gender (male/female)	Comorbid condition	Tumor information						Interval from postoperative to early recurrence, year	Treatment
							Cirrhosis	Maximum diameter (cm)	Number (≥2)	margin (smooth)	capsule	MVI	BCLC stage		
Li et al. (2022) (13)	R	January 2015-April 2018	T and V	302	T: 57.2 ± 9.8; V: 57.6 ± 10.2	245/57	216	≤5:170; >5:132	57	62	261	135	/	2	①
Zhang et al. (2019) (19)	P	June 2015-May 2018	T and V	155	50.35 ± 11.57	124/31	/	≤5:68; >5:87	54	62	118	62	0:13; A:34; B:68; C:40	1	①
Gao et al. (2022) (14)	R	January 2016-March 2017	T and V	472	T: 57 ± 12; V: 56 ± 11	386/86	/	T: 5.1 ± 3.7; V: 5.0 ± 3.9	/	/	/	176	0:99; A:373	2	①
Zhao et al. (2020) (27)	R	April 2007-April 2018	T and V	113	57.07 ± 10.17	92/21	86	≤5:88; >5:25	25	76	90	34	/	2	①
Zhang et al. (2021) (20)	R	June 2012-April 2018	T and V	132	54 (46–62)	114/18	/	2.00 (1.58–2.43)	26	/	/	/	/	2	②
Wang et al. (2022) (21)	R	January 2012-December 2017	T and V	190	54.86 ± 9.03	163/27	119	2.27-4.50	/	145	113	58	/	2	①
Chong et al. (2021) (22)	R	March 2012-June 2018	T and V	323	/	280/43	207	≤5:305;>5:18	54	171	/	88	0:96; A:195; B:22	2	①
Wang et al. (2022) (23)	R	January 2012-December 2017	T and V	170	/	146/24	102	2.20-4.60	/	/	/	/	/	2	①
Ren et al. (2022) (28)	R	June 2014-December 2017	T and V	270	/	226/44	179	2.30-6.70	33	/	/	30	/	1	①
Zhao et al. (2022) (7)	R	December 2013-July 2019	T and V	151	/	127/24	/	/	/	/	/	/	/	1	③
Ning et al. (2020) (24)	R	February 2012-August 2016	T and V	325	53.23 ± 9.79	292/33	303	≤5:150;>5:175	57	/	68	48	/	1	①
Shan et al. (2019) (25)	R	January 2010-September 2015	T and V	156	T:53.2 ± 12.4; V: 55.4 ± 10.6	88/68	95	T: 4.2 ± 2.9; V: 3.9 ± 3.3	36	/	/	/	/	2	① or ②
Zhou et al. (2017) (26)	R	December 2004-April 2015	T and V	215	54 (rang 15–84)	183/32	87	≤5:106;>5:109	23	/	108	17	0:23; A:162; B:13; C:17	1	①
Wu et al. (2022) (9)	R	May 2013-March 2020	T and V	132	/	122/10	52	≤5:74;>5:58	13	69	81	60	0+A:119; B:13	1	①
Wang et al. (2022) (10)	R	2017-2021	T and V	175	55 (rang 31–72)	152/23	124	≤2:32;>2:143	0	91	/	/	0:32; A:143	2	①

R indicated retrospective; P indicated prospective; T indicated training set; V indicated validation set; ①indicated curative resection; ②indicated curative ablation; ③indicated liver transplantation.

SD, standard deviation; MVI, microvascular invasion; BCLC, Barcelona Clinical Liver Cancer.

. /, not provided.

**Table 2:** Methodology of the included studies.

First author and year	Imaging equipment	MRI equipment	Contrast agent	Radiomics features	MRI sequence	Radiomics features extracting software	Modeling method	ER-present	ER-absent	TP	FP	FN	TN
Li et al. (2022) (13)	MRI	1.5T MR: scannerSigna Excite HD (GE Healthcare, Milwaukee, WI, USA) with an 8-channel phased-array software coil	Gd-DTPA	853 radiomics features (16 shape features, 19 first order features, 74 texture features, and 744 wavelet features)	T2-WI, DWI (b-value= 800 s/mm2), AP, PVP	ITK-SNAP	LASSO regression	141	161	127	50	14	111
Zhang et al. (2019) (19)	MRI	3.0T MR: Magnetom Skyra (Siemens Healthcare, Erlangen,	gadoxetic acid-enhanced	385 radiomics features (histogram, texture features, form factors, grey-level co-occurrence matrix, and grey-level run-length matrix)	T2-WI, unenhanced T1-WI, AP, PVP, and HBP	ITK-SNAP	LASSO regression	75	80	69	44	6	36
Germany) with an 18-channel body array coil													
Gao et al. (2022) (14)	MRI	1.5T MR: [Aera (Siemens, Erlangen, Germany and Avanto (Siemens, Erlan_x0002_gen, Germany)] and 3.0T MR: Magnetom Verio (Siemens Medical Solution, Erlangen, Germany)	gadoxetic acid-enhanced	864 radiomics features	axial T2-WI/FS, DWI (b-value= 0, 500 s/mm2), ADC (b-value= 0, 500 s/mm2), pre-contrast phase, late AP, PVP, and DP	ITK-SNAP	LASSO regression	133	339	76	74	57	265
Zhao et al. (2020) (27)	MRI	1.5T or 3.0T MR: Signa, HDXT (GE Healthcare, Milwaukee, WI) with an 8channel phased array body coil	Gd-DTPA	1146 radiomics (42 first-order histogram features, 15 shape features, 335 second-order texture features and 754 Gaussian transform features	T1WI, opposed-phase T1WI, T2WI, DWI, and CE-MR (AP, PVP, and DP)	ITK-SNAP	Logistic regression	58	55	43	19	15	36
Zhang et al. (2021) (20)	MRI	1.5T MR: GE Signa HDxt and 3.0T MR: Siemens Trio Tim, GE Signa Pioneer, GE Discovery MR750w	gadoxetic acid-enhanced	1316 radiomic features (14 shape features, 18 first-order intensity statistics features, 75 texture features, 465 logarithmic features, and 744 wavelet features)	T2-WI/FS, T1-WI/FS, AP, PVP, and hepatocellular phase	ITK-SNAP	random survival forest	77	55	69	17	8	38
Wang et al. (2022) (21)	MRI	3.0 T MR: Signa HDx (GE Medical System, Milwaukee, WI, USA);	Gadodiamide	1,316 radiomics features (18 first-order histogram features, 89 texture features, 744 wavelet features, 279 local binary pattern features, and 186 Laplacian of Gaussian features)	T2WI/FS and three-phase DCE-MR (AP, PVP, and DP)	ITK-SNAP	LASSO regression	80	110	64	31	16	79
Discovery MR 750 (GE Medical System) with an 8-channel													
phased-array body coil													
Chong et al. (2021) (22)	MRI	1.5T MR: Magnetom Aera (Siemens Healthcare, Erlangen, Germany)	Gd-EOB-DTPA	2950 features (the first-order statistics, shape and size, Gray-Level Co-occurrence Matrix, Gray-Level Run-Length Matrix, Gray-Level Size-Zone Matrix, Gray-Level Dependence Matrix, and Neighboring Gray-Tone Difference Matrix)	T2WI/FS, DWI, dynamic T1-WI/FS [(pre- and post-CE (AP, PVP, TP, and HBP)]	ITK-SNAP	Logistic regression	91	232	60	6	31	226
Wang et al. (2022) (23)	MRI	3.0T MR: (Signa HDx, GE Medical Systems, Milwaukee, WI, USA; Discovery MR 750, GE Medical Systems)	Gadodiamide	1316 radiomics features (18 first-order histogram features, 89 texture features, 744 wavelet features, 279 local binary pattern features, and 186 Laplacian of Gaussian features)	Whole-tumor ADC	ITK-SNAP	LASSO regression	69	101	43	39	26	62
Ren et al. (2022) (28)	MRI	3.0T MR: (GE MR Signa HDX 3.0T, Siemen MR Skyra 3.0T, and GE discovery MR 750 scanners)	/	1197 radiomics features	T1-WI, T2-WI, DWI (b-values: 800 s/mm2)	ITK-SNAP	Logistic regression	48	222	38	59	10	163
Zhao et al. (2022) (7)	CT	Discovery 750 HD (GE Health_x0002_care, Chicago, IL, USA)	Omnipaque 350	1218 radiomics features (first-order features, shape-based features, texture-based features, and first-order features and texture features extracted from images filtered by logarithm and wavelet transformation)	/	3D slicer	Logistic regression	33	118	28	18	5	100
Ning et al. (2020) (24)	CT	GE LightSpeed VCT, GE Healthcare, USA or Discovery CT750 HD, GE Healthcare, USA or SOMATOM Defnition Flash, Siemens, Germany or Bril_x0002_liance 16, Philips, Netherlands	Ultravist 370	656 radiomics features (gray features, shape features, texture features, and wavelet features)	/	ITK-SNAP	Logistic regression	176	149	126	25	50	124
Shan et al. (2019) (25)	CT	64-detector row (Aquilion CXL, Toshiba Medical System, Tokyo, Japan) or 320-detector row CT machine (Aquilion One, Toshiba Medical Sys_x0002_tem, Tokyo, Japan)	/	1044 radiomics features (gray-level histogram texture, wavelet transformed texture, transformed matrix texture, and filter-transformed texture)	/	Artificial Intelligence Kit	LASSO regression	75	81	46	27	29	54
Zhou et al. (2017) (26)	CT	Lightspeed Ultra 8 (GE Healthcare, Hino, Japan); 64-slice LightSpeed VCT (GE Medical systems, Mil_x0002_waukee, WI), or Brilliance iCT 256 (Philips Healthcare, Cleveland OH, USA)	Ultravist 370	300 radiomics features (the gray-level histogram and the graylevel co-occurrence matrix)	/	MATLAB 2014Ra	LASSO regression	102	113	81	34	21	79
Wu et al. (2022) (9)	CT	Siemens SOMATOM	/	396 radiomics features (the histogram, form factor, gray-level co-occurrence matrix, Haralick features, run-length matrix, and gray-level size zone matrix)	/	ITK-SNAP	Logistic regression	64	68	49	16	15	52
Wang et al. (2022) (10)	CT	Discovery 750 HD (GE Medical, USA), SOMATOM Force (Siemens Medical, Germany), Brilliance ICT (PHILIPS, Netherlands), or Aquilian One 640 (Toshiba, Japan) CT systems	Iopromide 370	1691 radiomics features (morphological, first order, texture, filter transform, and wavelet transform features)	/	Siemens Syngo Research Frontier platform	LASSO regression	56	119	13	3	43	116

MRI, magnetic resonance imaging; CT, computed tomography; WI, weighted imaging; DWI, diffusion-weighted imaging; AP, arterial phase; PVP, portal venous phase; HBP, hepatobiliary phase; FS, fat-suppressed; ADC, apparent diffusion coefficient; DP, delayed phase; CE, contrast enhanced; LASSO, least absolute shrinkage and selection operator; ER, early recurrence; TP, true positive; FP, false positive; FN, false negative; TN, true negative. /, not provided.

### Quality assessment of literature

**Figure 2:**
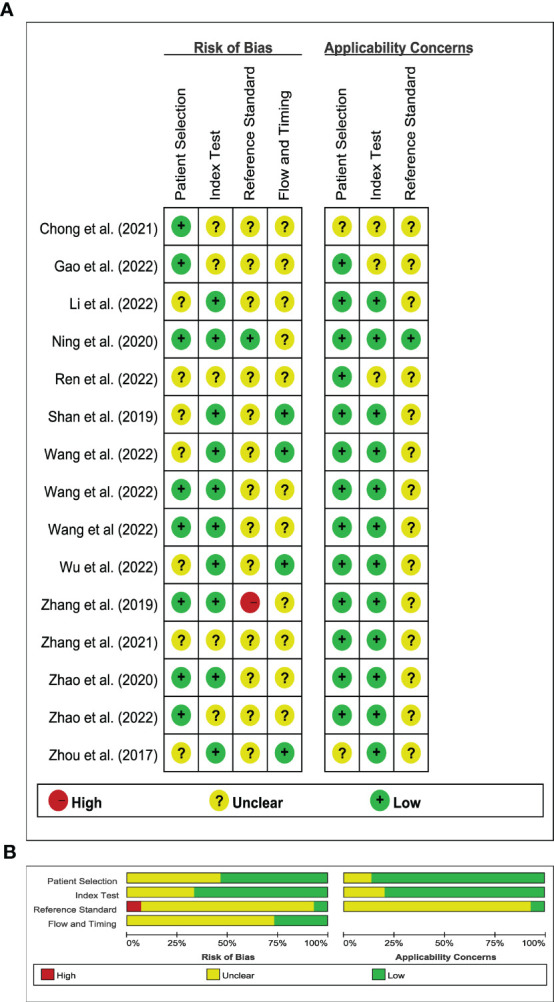
Stacked bar charts of the QUADAS-2 scale of methodological quality assessment. **(A)** Risk of bias and applicability concerns summary: review authors’ judgements about each domain for each included study; **(B)** Risk of bias and applicability concerns graph: review authors’ judgements about each domain presented as percentages across included studies.

**Figure 2** illustrated the results of the methodological quality assessment of the included studies based on the Quality Assessment of Diagnostic Accuracy Studies-2 (QUADAS-2) scale. Most studies were deemed to have a low to moderate risk of bias, with only mild concerns regarding their applicability. The included articles, as assessed by the Radiomics Quality Score (RQS) presented in **Table 3**, were determined to be of generally low quality.

**Table 3:** Radiomics quality scores.

Author	A	B	C	D	E	F	G	H	I	J	K	L	M	N	O	P	Total score
Li et al. (2022) (13)	0	1	0	1	3	1	0	1	1	1	0	2	2	2	0	0	15
Zhang et al. (2019) (19)	1	1	0	1	3	1	0	1	1	1	7	2	2	2	0	2	25
Gao et al. (2022) (14)	1	1	0	1	3	1	0	1	1	0	0	2	2	2	0	0	15
Zhao et al. (2020) (27)	0	1	0	1	3	1	0	1	1	1	0	2	2	2	0	0	15
Zhang et al. (2021) (20)	1	1	0	1	3	1	0	1	1	0	0	2	2	2	0	0	15
Wang et al. (2022) (21)	0	1	0	1	3	1	0	1	1	1	0	2	2	2	0	0	15
Chong et al. (2021) (22)	1	1	0	1	3	1	0	1	1	1	0	2	2	2	0	0	16
Wang et al. (2022) (23)	0	1	0	1	3	1	0	1	1	0	0	2	2	2	0	0	14
Ren et al. (2022) (28)	1	1	0	0	3	1	0	0	1	1	0	2	2	2	0	0	14
Zhao et al. (2022) (7)	1	1	0	1	3	1	0	1	1	1	0	2	2	2	0	0	16
Ning et al. (2020) (24)	1	1	0	1	3	1	0	1	0	0	0	2	2	2	0	0	14
Shan et al. (2019) (25)	1	1	0	1	3	1	0	0	1	1	0	2	2	2	0	0	15
Zhou et al. (2017) (26)	1	1	0	1	3	1	0	1	0	0	0	2	2	2	0	0	14
Wu et al. (2022) (9)	1	1	0	1	3	1	0	1	1	1	0	2	2	2	0	0	16
Wang et al. (2022) (10)	1	1	0	1	3	1	0	1	1	1	0	2	2	2	0	0	16

A,Image protocol quality; B, Multiple segmentations; C, Phantom study on all scanners; D, Imaging at multiple time points; E, Feature reduction or adjustment for multiple testing; F, Multivariable analysis with non-radiomics features; G, Detect and discuss biological correlates; H, Cutoff analyses; I, Discrimination statistics; J, Calibration statistics; K, Prospective study registered in a trial database; L, Validation; M, Comparison to gold standard; N, Potential clinical utility; O, Cost-effectiveness analysis; P, Open science and data.

### Heterogeneity analysis

The results of our study indicated high heterogeneity (Q= 211.53; I2 = 99%; 95%CI: 99-100), which required us to use the random-effects model to combine effect sizes. Additionally, the heterogeneity of sensitivity, specificity, PLR, NLR, diagnostic score, and DOR all exceeded 50%, making it necessary to account for this in our analysis. The threshold effect test showed the Spearman correlation coefficient to be 0.364 with P<0.182, implying no threshold effect and lending support to the combination of sensitivity and specificity.

### Pooled effect analysis

**Figure 3:**
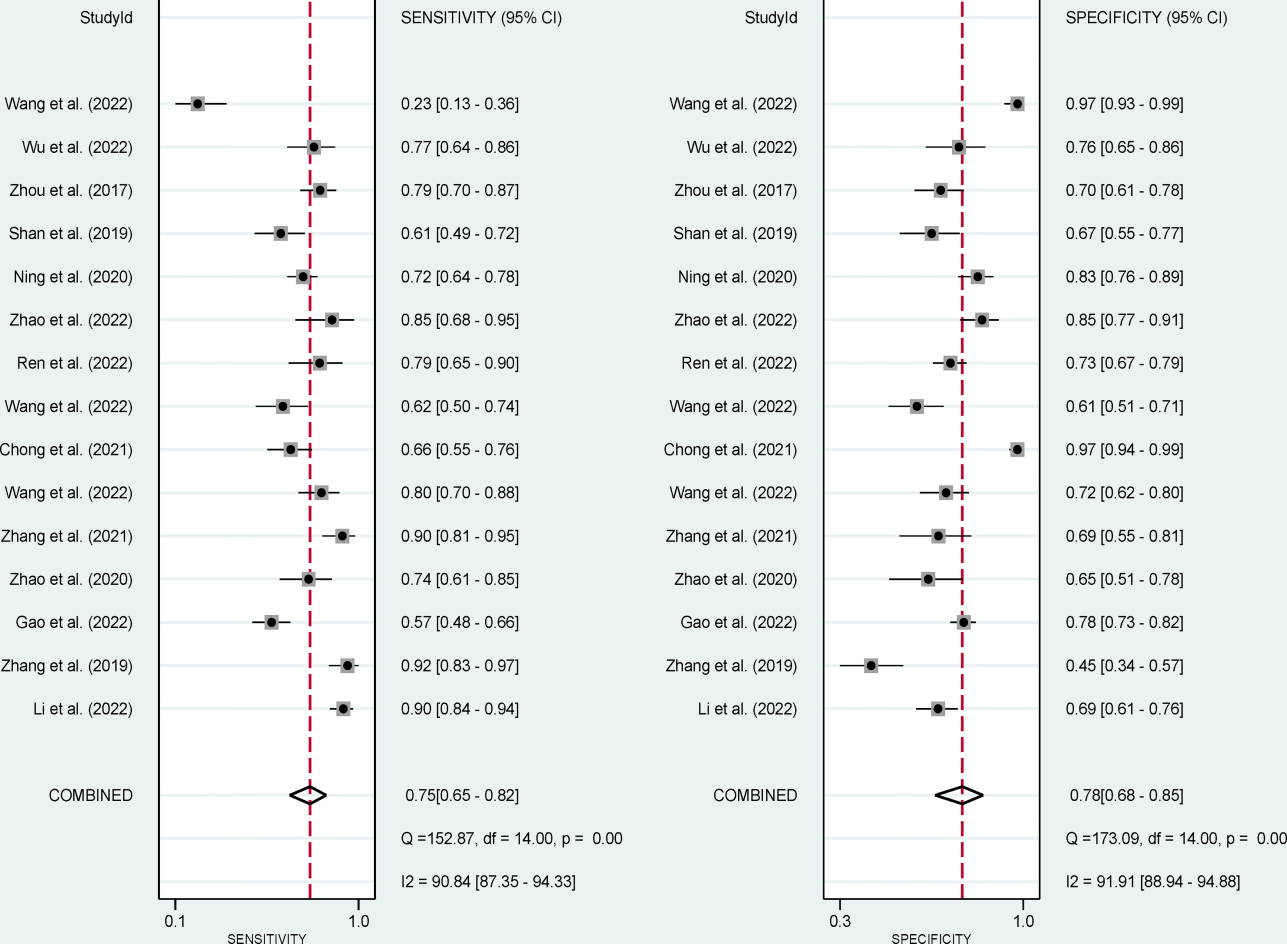
Forest plots show the performance estimates (sensitivity and specificity) of each study based on radiomics for the preoperative prediction of ER in HCC.

**Figure 4:**
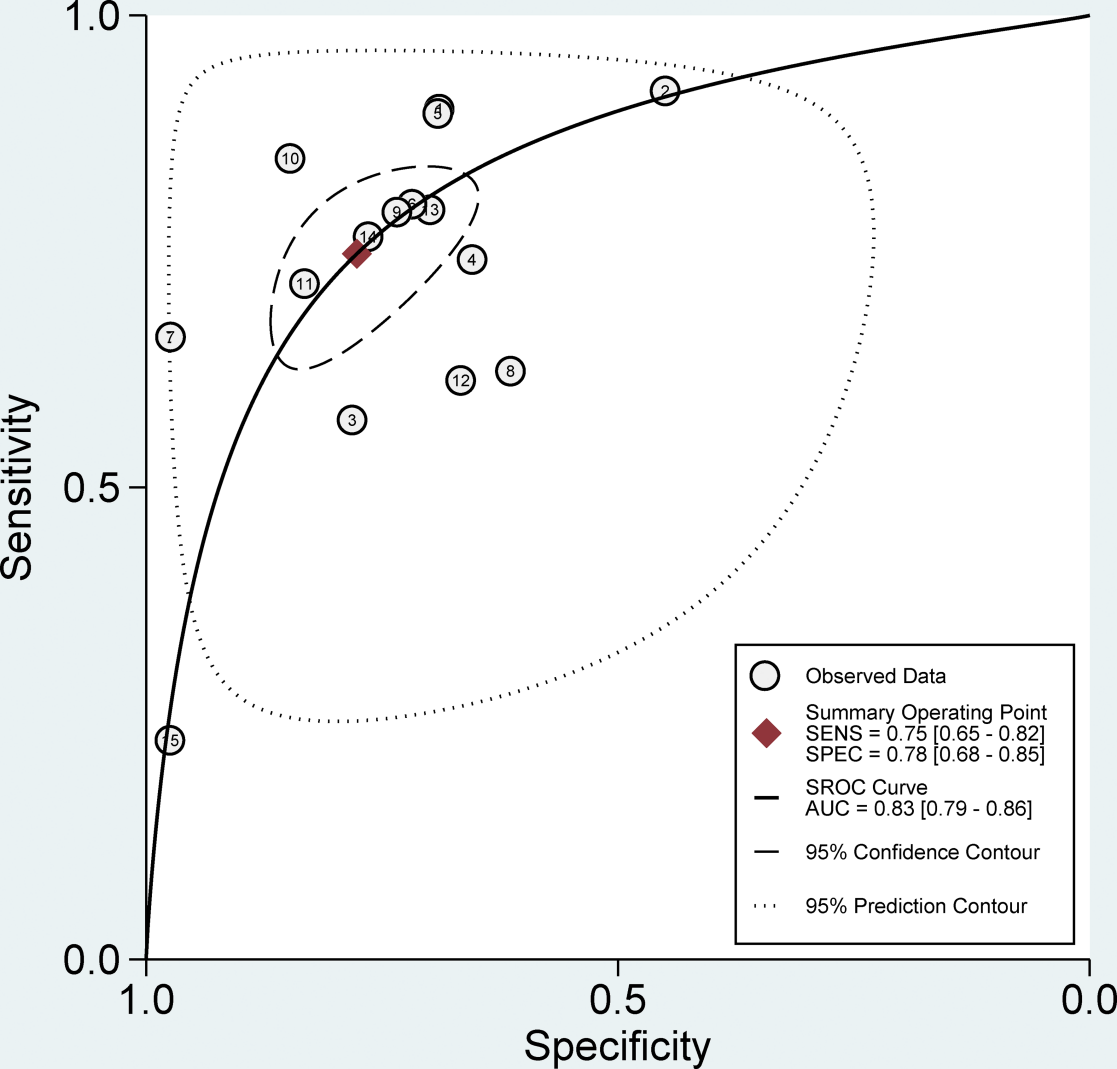
Forest plots show the performance estimates (SROC) of each study based on radiomics for the preoperative prediction of ER in HCC.

**Figure 5:**
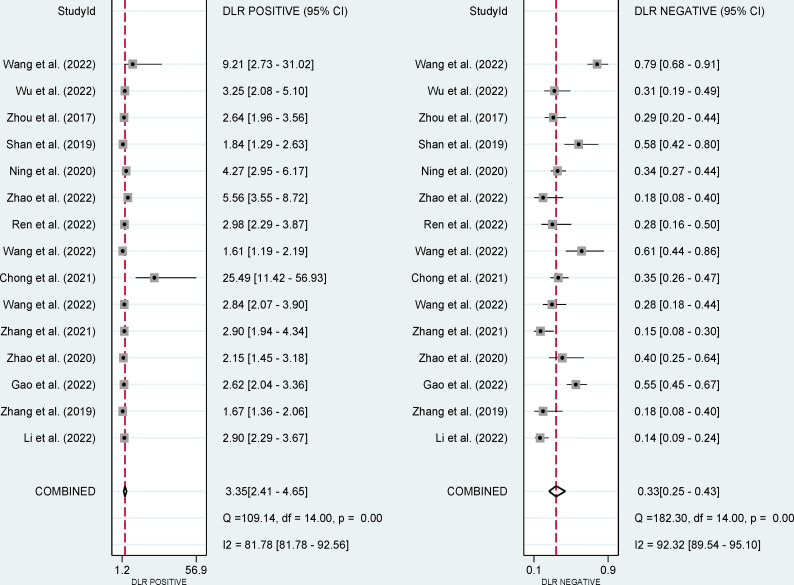
Forest plots show the performance estimates (PLR and NLR) of each study based on radiomics for the preoperative prediction of ER in HCC.

**Figure 6:**
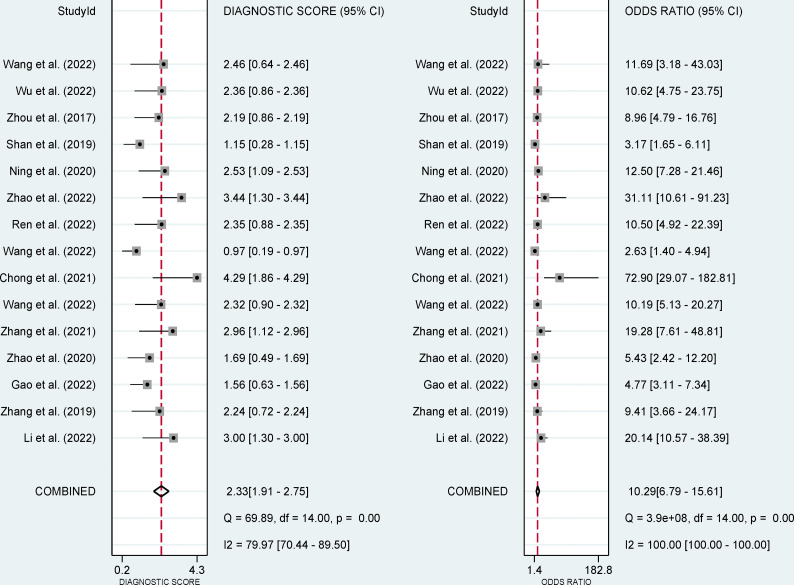
Forest plots show the performance estimates (diagnostic score, and DOR) of each study based on radiomics for the preoperative prediction of ER in HCC.

**Figure 7:**
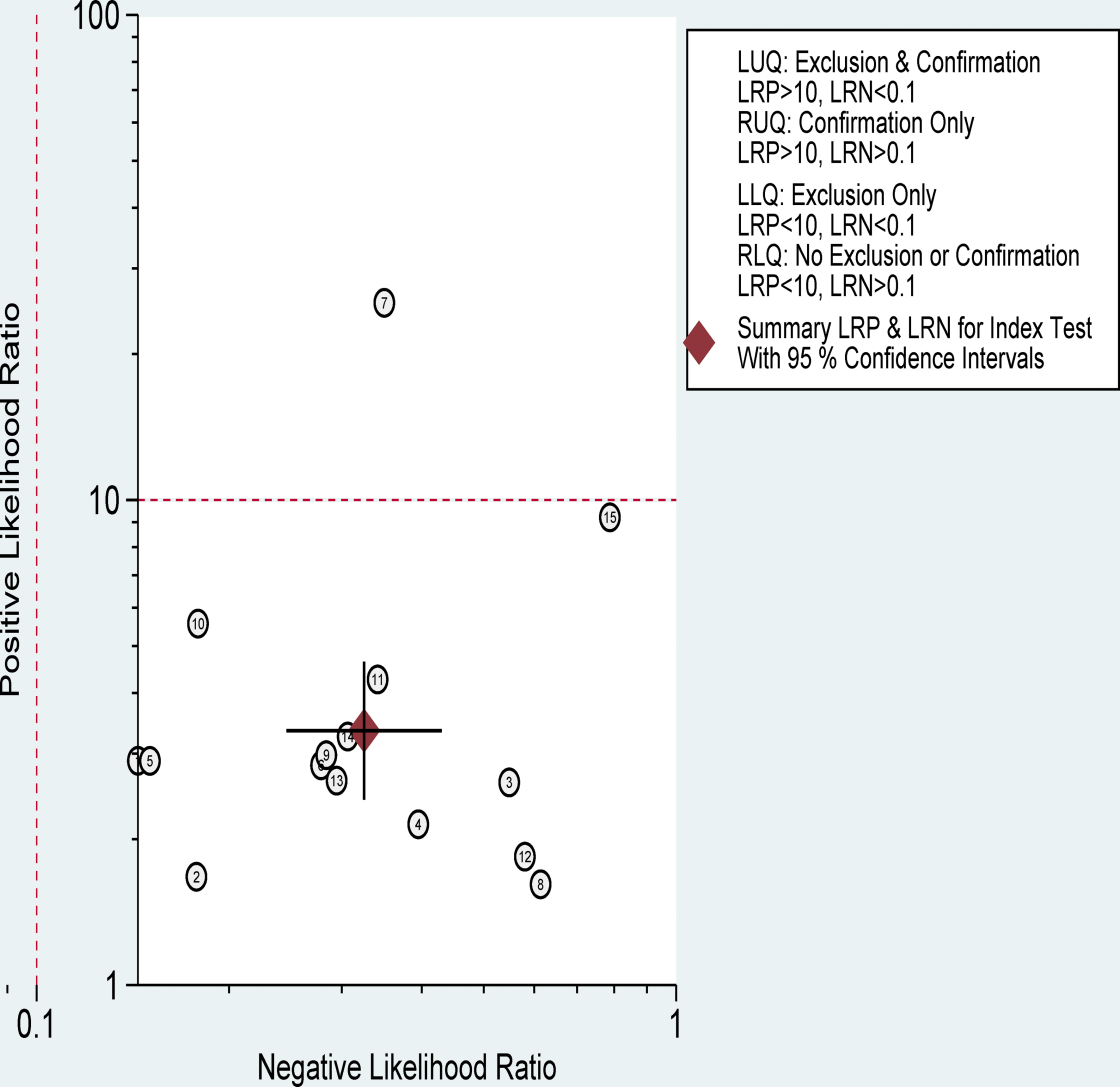
Distribution scatter diagram of the likelihood ratio (LR+/LR-) of each study and combined estimated value.

The pooled sensitivity, specificity, and AUC were 75% (95% CI: 65-82), 78% (95% CI: 68-85), and 83% (95% CI: 79-86), respectively (**Figures 3**, **4**). The combined PLR, NLR, diagnostic score, and DOR were 3.35 (95% CI: 2.41-4.65), 0.33 (95% CI: 0.25-0.43), 2.33 (95% CI: 1.91-2.75), and 10.29 (95% CI: 6.79-15.61), respectively (**Figures 5**, **6**). The scatter plot of the likelihood ratios showed that the pooled estimates with 95%CI were located in the lower right quadrant, suggesting that the combined accuracy of CT-based radiomics for diagnosing ER was moderate (**Figure 7**).

### Fagan nomogram analysis

**Figure 8:**
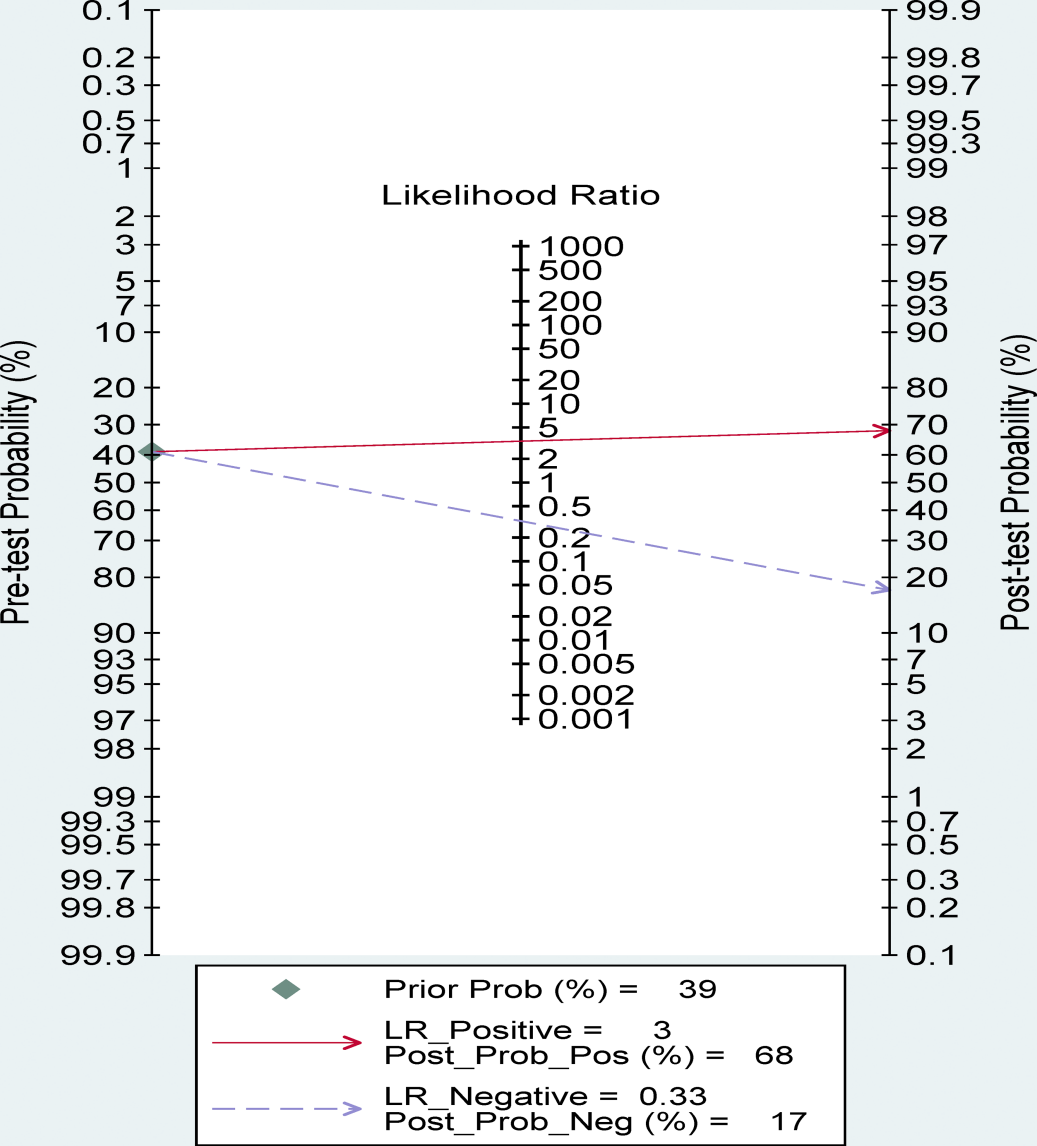
Fagan nomogram of radiomics for the preoperative identification of ER in HCC.

A 39% predicted probability was assessed to simulate a clinical situation, resulting in a posttest probability of 68% for a positive test result, and the negative posttest probability was 17% (**Figure 8**).

### Publication bias

**Figure 9:**
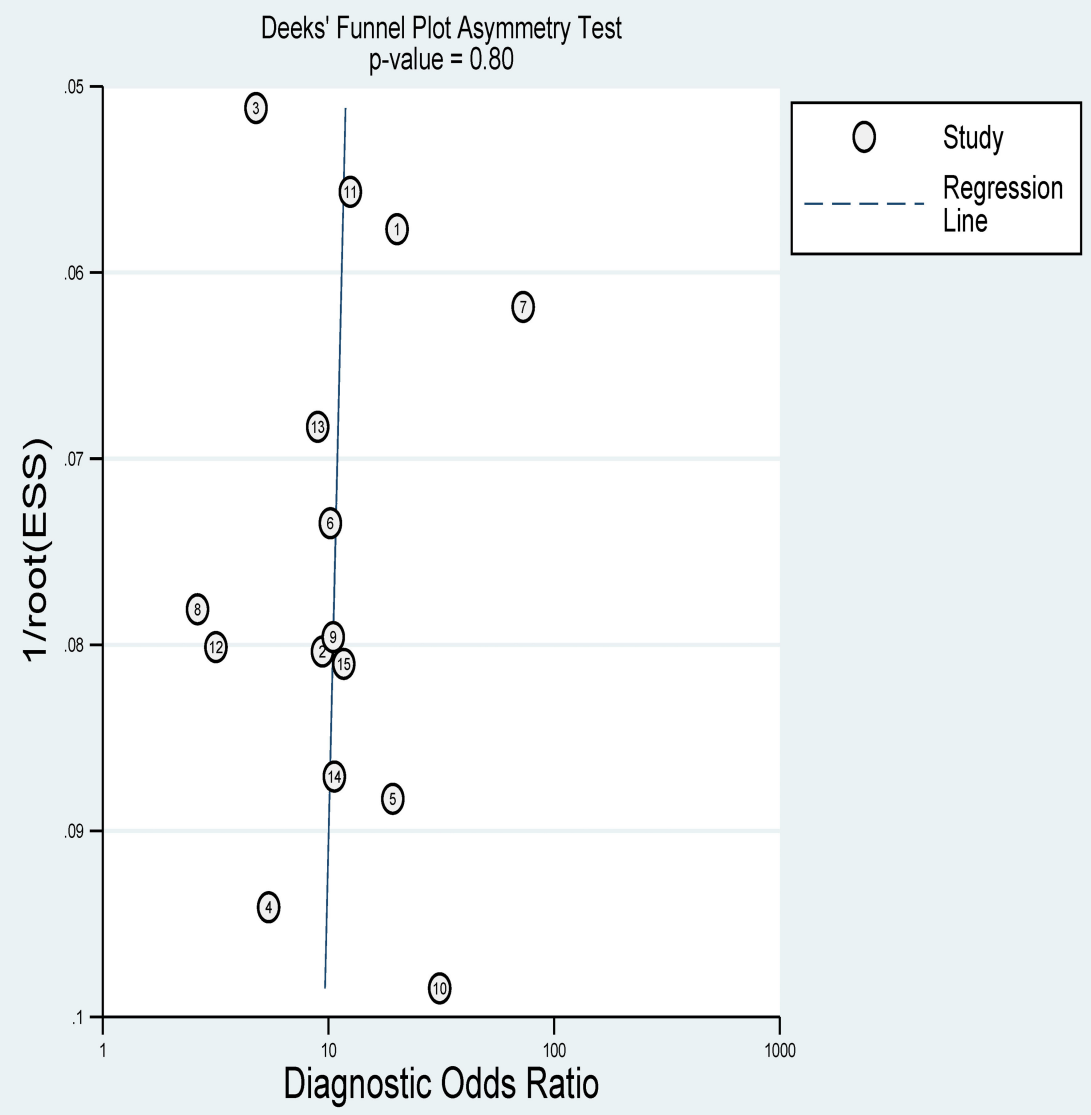
Deek funnel plot showing publication bias.

The Deek funnel plot showed a slope coefficient of 0.80, indicating that there was no publication bias in the included studies (**Figure 9**).

### Meta-regression and subgroup analysis

The forest plots indicated high heterogeneity with I^2^ values > 50% for sensitivity (I²=91%; 95% CI: 87-94, *P*< 0.001) and specificity (I²=92%; 95% CI: 89-95, *P*< 0.001). To identify the source of heterogeneity, we performed univariable meta-regression analysis. **Table 4** showed the results of univariable meta-regression and subgroup analyses to explore the influence of sample size, imaging equipment, modeling method, radiomics features extracting software. The results showed imaging equipment contributed to the heterogeneity in the specificity analysis (*P*= 0.03).

**Table 4:** Univariable meta-regression and subgroup analyses.

Parameter	Category	No. of Studies	Sensitivity (95%CI)	*P_1_*	Specificity (95%CI)	*P_2_*
Sample size	≥200	6	76 [63-88]	0.25	81[70-93]	0.50
	<200	9	74 [63-85]		74 [63-86]	
Modeling method¶	LASSO regression	8	71[59-83]	0.11	74 [61-86]	0.03
	Logistic regression	6	76 [63-88]		83[73-94]	
Imaging equipment	MRI	9	79 [70-88]	0.75	73 [62-85]	0.03
	CT	6	67 [53-82]		83 [72-94]	
Radiomics features extracting software	ITK-SNAP	11	78 [70-86]	0.86	75 [64-85]	0.05
	Other	4	63 [44-82]		84 [72-97]	

¶ one study used random survival forest as a modeling method.

In terms of imaging equipment, CT (n=6) had a higher specificity (83%; 95% CI: 72-94) than MRI (n=9) (73%; 95% CI: 62-85) (*P*= 0.03). However, the sensitivity [(67%; 95CI%: 53-82) vs. (79%; 95CI%: 70-88)] was basically equivalent for both (*P*= 0.75).

In terms of sample size, modeling method, radiomics features extracting software, the sensitivity (*P*= 0.25, 0.11, 0.86, respectively) were basically equivalent for both.

## Discussion

To our knowledge, this is the first study to investigate the predictive performance of preoperative CT/MRI-based radiomics for ER in HCC. Our meta-analysis showed moderate pooled sensitivity, specificity, and AUC were 75% (95% CI: 65-82), 78% (95% CI: 68-85), and 83% (95% CI: 79-86), respectively, which demonstrated radiomics has the potential to preoperatively differentiate ER for HCC. The confirmation of this evidence will help ER patients to develop the best postoperative diagnosis and treatment strategy, which plays a critical role in individualized risk stratification and further treatment guidance. For example, if preoperative radiomics highly suggests the possibility of future ER, more frequent follow-up should be taken for this population, the activity of the original tumor lesion should be closely monitored, and corresponding measures can be taken early to intervene, which is of great clinical significance for prolonging the survival of patients and improving long-term prognosis (11).

The likelihood ratio and the probability after the test also provide us with important information about the likelihood that a patient with a positive or negative test actually has ER or not. Our analysis found a PLR of 3.35 indicates that the test is 3.35 times more likely to correctly judge a positive result than to misjudge a positive result, resulting in a 68% post-test probability of a positive test result. Similarly, an NLR value of 0.33 indicates that the test is 0.33 times more likely to misjudge a negative result than to correctly judge a negative result, resulting in a 17% probability of a negative test result. These results further suggest that radiomics has important clinical value in preoperative evaluation of the occurrence of ER in HCC follow-up.

Although the test for heterogeneity showed significant variability among the included studies, the threshold effect test, measured by the Spearman correlation coefficient (0.364, P=0.182), indicated that the heterogeneity was not arise from threshold effects. Therefore, we performed meta-regression and subgroup analyses to try to explore possible sources of heterogeneity. Due to the limited number of included studies (n=15), we performed only univariate rather than multivariate meta-regression analyses. We used five key factors for subgroup analyses. Then, we performed subgroup analysis to compare the diagnostic performance of radiomics based on different imaging equipment. The results showed the sensitivity of CT and MRI was comparable. However, CT (n=6) has higher specificity than MRI (n=9) in predicting ER. Our comprehensive literature search failed to identify any studies that directly compared the performance of CT and MRI in predicting ER. This may be attributed to the fact that the majority of the literature reviewed consisted of diverse MRI sequence compositions with significant variations in sensitivity and specificity depending on the sequence selected (e.g., Zhang et al. used significantly fewer sequences than Chong et al.) (19, 22). However, CT uses radiomics of fixed parameters (enhanced arterial phase combined with venous phase) to predict ER, and imaging parameters are more uniform than MRI. Moreover, MRI image acquisition is more technically complex and sensitive to artifacts and image quality inhomogeneity in the diagnosis of HCC compared to CT. As such, patients with poor breath-holding ability or ascites may experience difficulties during MRI scanning, leading to lower quality images (29). Consequently, this may result in radiomics models failing to provide accurate and relevant imaging biomarker information for predicting ER of HCC. The pooled results which favored CT over MRI are not conclusive. Prospective, large-scale, and multi-center studies are needed to establish the superiority of one imaging method over the other. Although there is significant heterogeneity in sensitivity, we found no source of heterogeneity by regression analysis. We attempted to exclude the study by Wang et al. (10) due to its low sensitivity, but heterogeneity remained at 91.16% (95% CI: 87.56-94.76) (results not listed).

Recently, radiomics has developed rapidly and has been widely used in the study of tumors in different systems, including HCC. In recent years, with the application of the theoretical system and technical framework of radiomics in the study of ER of HCC, the prediction performance has also tended to be stable. However, there are still challenges that need to be addressed before its application in clinical practice. The lack of standardization in imaging equipment and acquisition parameters makes it difficult to uniformly analyze radiomics features, which may affect the practicality and feasibility of the model. In the future, a possible solution is to integrate the features extracted from both CT and MRI imaging methods may create a new breakthrough in radiomics in the prediction of ER of HCC. Establishing a comprehensive HCC image database and enabling data sharing is crucial to train and validate predictive models that simulate the real state and reduce non-biological differences.

The integration of radiomics and multi-omics data may lead to the emergence of multi-omics artificial intelligence, which can play a greater role in personalized medicine *via* non-invasive and personalized methods for assessing the occurrence, development, and prognosis of HCC. Furthermore, intelligent segmentation of imaging lesions will be a focus of future radiomics research, and potential correlations between radiomics features and biological characteristics can be further clarified through radiogenomics-related index features. However, the complex relationship between radiomics features and biological behavior may be difficult to fully eliminate.

Our meta-analysis had some limitations. Firstly, most studies (n=14) we included were retrospective, and patient selection may introduce some inherent bias. Secondly, all included studies were from China. Therefore, there are differences in disease background due to different regions, countries, ethnicities, etc., and HCC is highly heterogeneous, which may affect the general applicability of the results in clinical practice. Thirdly, not all included studies used uniform pathological biopsy to confirm the diagnosis of ER, and the diagnosis was interspersed with imaging (although only for those with typical radiographic findings), and the interpretation of imaging images may depend on the reader’s experience, which may partly explain differences in sensitivity and specificity between studies. Fourthly, we only searched the foreign language databases, which may introduce publication bias. However, we believe that this bias should be relatively small, as no publication bias was observed in this study. Moreover, the heterogeneity in imaging modality, feature extraction among related studies needs to be carefully considered as radiomics is a platform method rather than a single marker. Finally, although CT/MRI radiomics models are helpful in identifying ER, the modeling methods used may affect the predictions of radiomics analysis. Each study developed a different radiomics model, so the current meta-analysis does not clarify a clear modeling approach to diagnose ER.

## Conclusion

In summary, this meta-analysis suggested that preoperative CT/MRI radiomics is a promising noninvasive predictive method with moderate ER recognition performance and is a crucial guide for clinical follow-up planning of postoperative HCC patients. We believe that prospective, large-scale, and multicenter studies utilizing multimodal radiomics methods will improve the predictive power of ER in the future.

## Data availability statement

The original contributions presented in the study are included in the article/supplementary material. Further inquiries can be directed to the corresponding author.

## Author contributions

Study concepts and design: HT, YX, and ZW. Literature research: HT and YX. Data collection: HT and YX. Data analysis: HT and YX. Manuscript writing: HT. Manuscript review: ZW. All authors contributed to the article and approved the submitted version.
